# Laryngitis Catarrhalis—Pseudo Croup

**Published:** 1879-12

**Authors:** W. Salm

**Affiliations:** Atlanta, Ga.


ATLANTA
Medical and Surgical Journal.

VOL. XVIII.] DECEMBER—1879. [No. ~~8~~ 9

### Original Communications.

---

### LARYNGITIS CATARRHALIS—PSEUDO CROUP.

BY W. SALM, M.D., ATLANTA, GA.

Pseudo or spasmodic croup is the result of catarrhal inflammation of the larynx and trachea, and consists of spasm of one or both of these portions of the air passage. Symptoms equal in violence to those found in true membraneous croup are produced by the spasm, but not of long duration; the paroxysms lasting only for fifteen to thirty minutes, if proper means for relief be resorted to. These attacks of difficult breathing may occur several times during the night, but generally only once.

The child who, during the day, shows symptoms of slight cold, is cheerful when put to bed, but arouses the mother by a hoarse, ringing cough and difficult breathing. Insufficient amount of air reaches the lungs to oxyginise the blood, and with the distressing effort to breathe is seen blueish colored lips and fingers; the little sufferer tossing from side to side, and rising suddenly in the agony of dyspnœa reaching for breath. Very simple means, sometimes, relieves the patient. Inhalation of warm vapor of vinegar, or even water, with frequent small drinks of warm tysans, gives complete comfort for the time, and often for the whole night. Occasionally, however, the spasm is more intractable. Nausea and vomiting from ipecac or tartarized antimony, become necessary in such cases, and the syrup of ipecac should be given in teaspoonful doses, every half hour, till complete relaxation is induced, when the spasm subsides and the patient once more breathes easily. A more violent, and perhaps better, remedy is found in the compound syrup of squills, sometimes called Cox's hive syrup. This, in the dose of a teaspoonful, produces deathly nausea and the most complete relaxation. The preparation, in the dose advised, contains one-eighth of a grain of tartar emetic, which is not only a nauseant but sedative to the circulation also, thereby allaying abnormal arterial excitement.

The day succeeding an attack of spasmodic or false croup is often spent comfortably by the patient, but at about the same hour of the preceding, a paroxysm may be expected the following night. Forewarned, the parents should be forearmed, and give some warm relaxing drinks or nauseating remedy to ward off an attack, particularly if sonorous, croupy breathing or cough be heard during the night. The disease under consideration rarely proves fatal, but so nearly resembles true or pseudo-membranous croup in the symptoms witnessed during a paroxysm, that alarm is always felt; and in fact the dyspnœa is so distressing that great solicitude is felt by the physician as well as parents, even when the condition is believed to be spasmodic in character. The suddenness of attack will generally be sufficient evidence of the paroxysmal variety, but if there be doubt as to the grave character of disease, the prompt relief afforded by a nauseant, which is by no means objectionable in pseudo-membranous croup, will at once settle the question. If pseudo croup exists, prompt relief will generally be afforded, but if pseudo-membranous croup is in hand, slight and temporary relief only may be expected.

True membranous croup is one of the most formidable diseases with which the physician meets, and his skill and energies will be exhausted generally without preventing a fatal termination. The usual relaxing, sedative and mercurial plan failing to succeed, laringotomy has been resorted to in Atlanta, but only with the addition of a few days to the tenure of life. The mechanical irritation of the tube inserted, so aggravates the existing inflammation that although respiration is free and the blood properly aeriated, the patient finally succumbs to the ordinary results of acute inflammation.

Reasoning from the known beneficial effects of carbolic acid in diphtheretic inflammation, in which the pseudo-membrane is readily dissolved, we venture the declaration that the false membrane in true or pseudo-membranous croup may also be removed if the remedy in sufficient concentration could be applied to it. Could not the vapor of this remedy be inhaled so as to impress the larynx and trachea sufficiently to accomplish this very desirable object? We hope some one will test its virtues in this respect.

